# Markers for Routine Assessment of Fatigue and Recovery in Male and Female Team Sport Athletes during High-Intensity Interval Training

**DOI:** 10.1371/journal.pone.0139801

**Published:** 2015-10-07

**Authors:** Thimo Wiewelhove, Christian Raeder, Tim Meyer, Michael Kellmann, Mark Pfeiffer, Alexander Ferrauti

**Affiliations:** 1 Faculty of Sport Science, Ruhr-University, Bochum, Germany; 2 Institute of Sports and Preventive Medicine, Saarland University, Saarbrücken, Germany; 3 School of Human Movement Studies and School of Psychology, The University of Queensland, Brisbane, Australia; 4 Institute of Sports Science, Johannes-Gutenberg University, Mainz, Germany; University of the Balearic Islands, SPAIN

## Abstract

**Aim:**

Our study aimed to investigate changes of different markers for routine assessment of fatigue and recovery in response to high-intensity interval training (HIIT).

**Methods:**

22 well-trained male and female team sport athletes (age, 23.0 ± 2.7 years; V̇O_2max_, 57.6 ± 8.6 mL·min·kg^−1^) participated in a six-day running-based HIIT-microcycle with a total of eleven HIIT sessions. Repeated sprint ability (RSA; criterion measure of fatigue and recovery), countermovement jump (CMJ) height, jump efficiency in a multiple rebound jump test (MRJ), 20-m sprint performance, muscle contractile properties, serum concentrations of creatinkinase (CK), c-reactive protein (CRP) and urea as well as perceived muscle soreness (DOMS) were measured pre and post the training program as well as after 72 h of recovery.

**Results:**

Following the microcycle significant changes (*p* < 0.05) in RSA as well as in CMJ and MRJ performance could be observed, showing a decline (%Δ ± 90% confidence limits, ES = effect size; RSA: -3.8 ± 1.0, ES = -1.51; CMJ: 8.4 ± 2.9, ES = -1.35; MRJ: 17.4 ± 4.5, ES = -1.60) and a return to baseline level (RSA: 2.8 ± 2.6, ES = 0.53; CMJ: 4.1 ± 2.9, ES = 0.68; MRJ: 6.5 ± 4.5, ES = 0.63) after 72 h of recovery. Athletes also demonstrated significant changes (*p* < 0.05) in muscle contractile properties, CK, and DOMS following the training program and after the recovery period. In contrast, CRP and urea remained unchanged throughout the study. Further analysis revealed that the accuracy of markers for assessment of fatigue and recovery in comparison to RSA derived from a contingency table was insufficient. Multiple regression analysis also showed no correlations between changes in RSA and any of the markers.

**Conclusions:**

Mean changes in measures of neuromuscular function, CK and DOMS are related to HIIT induced fatigue and subsequent recovery. However, low accuracy of a single or combined use of these markers requires the verification of their applicability on an individual basis.

## Introduction

High-intensity interval training (HIIT), involving short to long (~5–300 s) intensive work intervals interspersed by active or passive recovery periods, is frequently used in training programs of competitive team sport athletes. This type of intermittent training was shown to improve cardiovascular and metabolic determinants, allowing players to sustain intense phases during the game for longer durations and also to recover from it more rapidly [1, 2]. Additionally, HIIT induces similar adaptations with significant lower training volumes compared to traditional endurance training [3, 4]. This is the main rationale behind its application in team sport conditioning programs, since the complex profile of demands requires that various conditional abilities as well as technical and tactical elements need to be considered and, consequently, the timeframe to improve endurance performance is limited.

However, as a result of high metabolic and neuromuscular demands, HIIT is also accompanied with acute feelings of fatigue [5]. Howatson and Milak [6] have shown that even one single team sport specific HIIT session leads to a significant increase in muscle damage and muscle soreness in the days following the exercise bout. Although effective training programs intend functional overreaching, excessive overload with insufficient recovery should be avoided [7]. If the balance between training stress and recovery is inadequate over a prolonged period, the athlete will experience decreases in performance and a state of overtraining may develop [7]. During in-season training, the challenge for coaches and athletes is to determine the point at which HIIT may negatively affect the performance in upcoming competitions. Therefore, the routine assessment of fatigue and recovery during HIIT is important to improve individual training prescriptions and to ensure competition readiness [8].

Fatigue and recovery is characterized by a combination of several factors involving mechanisms from the central nervous system to the muscle cell itself. In this regard, a change in the players’ specific on-court performance represents the most relevant marker for differentiation between fatigued and recovered athletes. However, the majority of field test recommendations for standardized performance measurements in team sports are physically demanding and induce additional fatigue [9, 10]. Consequently, a variety of other surrogate markers (e.g., subjective, biochemical, neuromuscular, and performance markers) are frequently used in science and practice in order to track the fatigue and recovery process [9]. The daily determination of a wide range of these markers established in endurance sports (e.g., heart rate variability or several markers in the blood), however, seems to be inadequate and difficult to control under the typical team sport surrounding. Therefore, practical parameters that are determined at rest or during low metabolic and neuromuscular demands, without disturbing the training process, are preferred in team sports for the routine assessment of fatigue and recovery [11].

Tools that meet these criteria and that have been proposed in the literature are subjective markers (e.g., delayed onset muscle soreness), neuromuscular performance tests (e.g., jumps), muscle contractile markers (e.g., measured via Tensiomyography) and routine capillary blood parameters (e.g., creatinkinase) [9, 12–14]. However, there is still no consensus regarding the usefulness of these simple tests for the routine assessment of fatigue and recovery in team sport athletes during and after HIIT [7, 9, 11]. Thus, the aim of this study was to investigate the accuracy of the aforementioned markers to reflect changes in fatigue and recovery in response to a six-day HIIT program, designed to induce a temporary functional overload, as well as after 72 h of recovery in male and female team sport athletes. We hypothesized that the training program leads to relevant changes in team sport specific performance and in related variances in markers of fatigue and recovery.

## Materials and Methods

### Participants

A total of 22 (11 males and 11 females) healthy and well-trained team sport athletes (i.e., soccer, basketball, handball) took part in this study. The baseline physical characteristics of the athletes are shown in Table 1. The mean training frequency of the athletes was 5.7 d·week^−1^ with a mean training volume of 2.5 h·day^−1^. After being informed about the exercise protocols and all possible risks associated with participation in the investigation, subjects gave written consents to participate in all procedures. Normal ECG and the absence of cardiovascular, pulmonary and orthopedic diseases were confirmed in a preliminary health examination. Additionally, athletes had to meet two inclusion criteria: minimal performance in the 30–15 Intermittent Fitness Test (30-15_IFT_) of at least 16 km∙h^−1^ for women or 19 km∙h^−1^ for men and at least five years of specific team sport training experience. Initially, 24 athletes from different regional teams were evaluated for possible participation in the study, of which two failed to fulfill the inclusion criteria. The study was approved by the ethic committee of the Medical Faculty of the Ruhr-University Bochum and performed according to the Declaration of Helsinki.

**Table 1:** Baseline physical characteristics of the athletes.

	Age (yrs)	Height (cm)	Body mass (kg)	Body fat (%)	V̇O_2max_ (mL·min·kg^−1^)
Overall (n = 22)	23.0±2.7	176.6±7.6	69.5±7.3	17.9±5.8	57.6±8.6
Male (n = 11)	22.9±1.9	181.6±5.3	73.8±6.4	14.6±3.7	62.9±8.3
Female (n = 11)	23.0±3.4	171.6±6.0	65.2±5.5	21.1±5.9	52.2±4.8

Parameters are shown as mean ± SD.

### Experimental design

**Fig 1:**
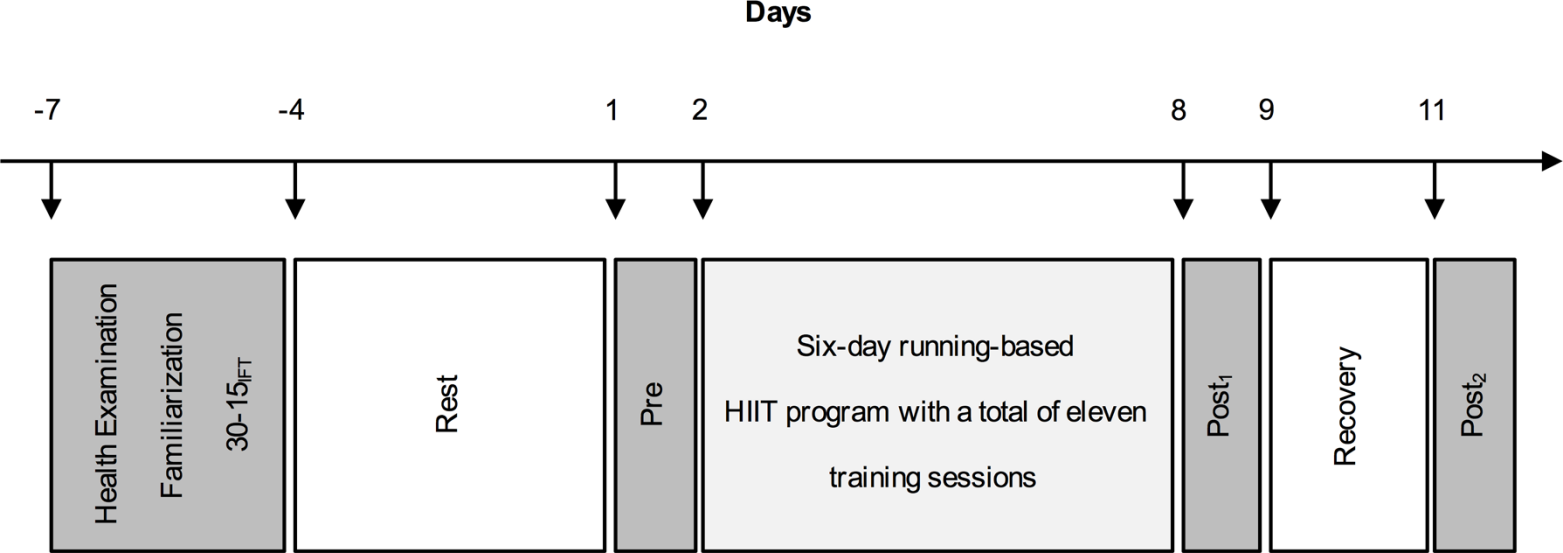
Study design. 30-15_IFT_ = 30–15 Intermittent Fitness Test, HIIT = high-intensity interval training.

A repeated measures study was used to examine the accuracy of markers of fatigue and recovery. The investigation lasted 18 days and was conducted in the athletes’ off-season period during which no additional club training took place. Seven days prior to the HIIT-program all athletes came to the laboratory for a preliminary health examination to exclude contraindications to participation in this study (e.g., cardiovascular, pulmonary, or orthopedic diseases), to obtain data on anthropometrical characteristics and to determine V̇O_2max_. After familiarization with performance tests to minimize any learning effect, participants completed the 30-15_IFT_ on a second preliminary examination day followed by four days of rest. Athletes were then examined at baseline (pre), after completing a six-day training program of HIIT (post_1_), and following a 72 h recovery period (post_2_), in which no training was allowed (Fig 1).

On all testing days (pre, post_1_, post_2_), repeated sprint ability (RSA) was assessed on a nonmotorized treadmill (NMT), which was defined as an important marker of team sport specific performance and as criterion measure of fatigue and recovery. RSA test has been shown to be closely associated with competitive performance in team sport athletes [15–17] and to be highly reproducible (coefficient of variation (CV) of about 2.5% for velocity) [18, 19]. In addition, prior to the RSA test, perceived muscle soreness, muscle contractile properties, blood parameters as well as jump and linear sprint performance were determined (in this order) as surrogate markers of fatigue and recovery pre, post_1_, and post_2_. All measures were taken at the same time of day for each individual on each occasion. Time between jump tests and linear sprint test as well as between linear sprint and RSA test was 30 min and 120 min, respectively. Prior to all performance tests a standardized warm-up was conducted.

Participants were instructed to maintain their normal dietary intake and to refrain from nutritional supplements and alcohol intake during the experimental period. In this regard, athletes were verbally questioned before each testing procedure as well as during the six-day training intervention to ensure that they had adhered to the dietary rules.

### Procedures

#### Incremental treadmill test

In order to determine V̇O_2max_, a progressive incremental exercise test on a motor driven treadmill (Ergo ELG2, Woodway GmbH, Weil am Rhein, Germany) was used. The treadmill test started with an initial velocity of 8 km∙h^−1^, increasing 2 km∙h^−1^ every 3 min with a constant incline of 0.5% until voluntary exhaustion. V̇O_2_ was continuously analyzed using a breath-by-breath gas collection system (ZAN600USB, nSpire Health GmbH, Oberthulba, Germany). The gas calibration was completed before the test day and the volume calibration was conducted before each test following the instructions provided by the manufacturer. The highest mean value for 30 s was defined as the V̇O_2max._

#### 30–15 intermittent fitness test

The test was conducted outdoors on a tartan track and consisted of 30 s shuttle runs interspersed with 15 s passive recovery periods. Speed was set at 8 km∙h^−1^ for the first 30 s run and was increased by 0.5 km∙h^−1^ every 45 s stage thereafter. The athletes had to run back and forth between two lines set 40 m apart at a pace dictated by an acoustic signal. The test ended when a player could no longer maintain the imposed running speed or when he was unable to reach a 3 m zone around each line at the moment of the audio signal for three consecutive times. The speed of the last completed stage achieved by the participants (V_IFT_) was used as an inclusion criteria and to calculate the interval intensity of the HIIT protocols applied in the six-day training program as described by Buchheit [20].

#### Repeated sprint ability test

The laboratory RSA test was performed on a Woodway nonmotorized treadmill (NMT) (Force 3.0, Woodway GmbH, Weil am Rhein, Germany) pre, post_1_, and post_2_. The experimental set-up of the test has previously been described by Oliver et al. [18]. The RSA test consisted of six 4 s maximal sprints from a standing position with 20 s passive recovery between sprints. The peak values attained in each sprint for velocity were recorded and mean peak values for velocity (MV) were calculated. For MV, the intraclass correlation coefficient (ICC) and the typical error (TE) were previously investigated by our research group and MV was considered to be highly reliable (unpublished results: MV (m·s^−1^), n = 17, ICC = 0.92, TE = 0.10, CV = 1.5%).

#### Jump and linear sprint tests

On each testing day (pre, post_1_, and post_2_), countermovement jumps (CMJ) and multiple rebound jump tests (MRJ) were performed on a contact platform (Haynl-Elektronik GmbH, Schönebeck, Germany) with hands placed on hips. For CMJ, participants dropped down to a self-selected level before jumping maximally. Flight time was used to calculate jump height [21]. Each subject performed two maximal CMJ and the mean height was calculated. For MRJ, participants were advised to perform repeated maximum vertical jumps for 15 s with reactive landing phases and ground contact times which should be as short as possible. Flight time and contact time were used to calculate the reactive strength index (RSI) for each jump by dividing the height jumped in meters by the time on the ground in seconds [22]. Based on the RSI, the five best jumps were selected and mean RSI was calculated for further analysis. The 20-m linear sprint was completed outdoors on a tartan track and sprint times were recorded using a wireless double-photocell system (Sportronic, Winnenden-Hertmannsweiler, Germany). Each sprint was initiated without a starting signal and from an individually chosen upright standing position 50 cm behind the first photocell. Participants performed two maximal sprints interspersed by 3 min of passive recovery and the mean sprint time was calculated for further analysis. Previously measured reliability scores for jump and linear sprint tests were regarded as highly reliable (unpublished results: CMJ (cm), n = 38, ICC = 0.92, TE = 1.86, CV = 3.7%; MRJ (RSI), n = 38, ICC = 0.91, TE = 0.13, CV = 4.0%; 20-m linear sprint test (s), n = 22, ICC = 0.95, TE = 0.06, CV = 1.8%).

#### Muscle contractile markers

For the non-invasive assessment of the contractile properties of knee extensor and flexor muscles, Tensiomyography (TMG) was used under laboratory conditions pre, post_1_, and post_2_. This technique produces radial displacement of the muscle belly in response to an electrical stimulus (around 100 mA) conducted through the underlying muscle tissue [13, 23]. These displacements are recorded at the skin surface using a spring loaded displacement sensor (TMG-BMC Ltd, Ljubljana, Slovenia). The sensor was positioned perpendicular to the thickest part of the muscle belly, which was established visually and through palpation during a voluntary contraction, and the self-adhesive electrodes were placed symmetrically approximately 5 cm away from the sensor. Once the exact position for the sensor and electrodes was found, it was marked with a dermatological pen and kept constant during the experimental period. Maximal radial muscle belly displacement (Dm) and contraction time between 10 and 90% Dm (Tc) of the rectus femoris (RF) and biceps femoris (BF) were measured through TMG. Reliability scores for Dm and Tc of the RF and BF were previously examined and considered as reliable (unpublished results: RF Dm (mm), n = 20, ICC = 0.92, TE = 1.00, CV = 9.3%; RF Tc (ms), n = 20, ICC = 0.94, TE = 1.90, CV = 4.9%; BF Dm (mm), n = 20, ICC = 0.95, TE = 0.90, CV = 10.4%; BF Tc (ms), n = 20, ICC = 0.91, TE = 5.60, CV = 8.7%).

#### Biochemical markers

Venous blood samples were collected on each testing day (pre, post_1_, and post_2_; between 8 and 10 a.m., and ~2 h after the athletes took a typical breakfast) from an antecubital arm vein of the right arm using a 20-gauge disposable Safety-Multifly^®^ needle (Sarstedt AG & Co, Nümbrecht, Germany) while the subject was in a supine position. Samples were collected into 7.5 mL serum gel tubes with clotting activator (Sarstedt AG & Co, Nümbrecht, Germany) and subsequently centrifuged at 3500 rpm for 15 min within 20 min after sampling. The resulting serum was separated from the other compounds, pipetted into micro tubes (Sarstedt AG & Co, Nümbrecht, Germany) and stored at -80°C. Later, routine techniques (UniCel^®^ DxC 600 Synchron^®^, Beckmann Coulter GmbH, Krefeld, Germany) were used for analysis of the concentration of creatinkinase (CK), c-reactive protein (CRP), and urea. The diagnostic laboratory used in this study held current quality assurance certification (Referenzinstitut für Bioanalytik, Bonn, Germany).

#### Subjective marker

Before all tests were performed on each testing day (pre, post_1_, and post_2_), athletes were asked to score on a visual analogue scale (VAS) the general amount of delayed onset muscle soreness (DOMS). The VAS, which has been shown to be reliable in previous research [24], consisted of a 100 mm line whose endpoints were labeled by “no pain” (left) and “unbearable pain” (right). Subjects had to draw a vertical line at a point on the line that represented their pain at the time of measurement best. The rating resulted from the distance in mm from the left border of the scale to the point marked [14].

### Training program

A six-day training intervention was designed to induce a functional overload while remaining tolerable for the athletes. The training program (exercise mode, number and duration of intervals and rest, intensity) consisted of 11 training sessions with an average training duration of 35 min per session (Table 2). To calculate training intensity, participants completed the 30-15_IFT_ as part of the preliminary examinations. All sessions were completed outdoors on a 400 m tartan track and preceded by a standardized continuous 10 min warm-up, consisting of 40 m shuttle runs at 60–70% HR_max_ followed by four 40 m acceleration sprints. To ensure that the intended training intensity was maintained by the athletes, all sessions were supervised and individually calculated running distances were controlled. Additionally, training loads were determined by multiplying the numerical score of the athletes`perception of effort, using a category-ratio RPE scale [25, 26], with the total exercise duration in min. Training loads were kept constant throughout the training period.

**Table 2:** Six-day high-intensity interval training program.

	Day 1	Day 2	Day 3	Day 4	Day 5	Day 6
	Straight-line runs	Straight-line runs	Straight-line runs		Straight-line runs	Straight-line runs
	4 x 4 min	7 x 2 min	4 x 4 min		4 x 4 min	7 x 2 min
**a.m.**	(r = 3 min)	(r = 2 min)	(r = 3 min)	Rest	(r = 3 min)	(r = 2 min)
	80% V_IFT_	85% V_IFT_	80% V_IFT_		80% V_IFT_	85% V_IFT_
	TL: 231 ± 59	TL: 236 ± 48	TL: 210 ± 63		TL: 217 ± 71	TL: 256 ± 56
	Straight-line sprints	40m-shuttle runs	Straight-line sprints	40m-shuttle runs	Straight-line sprints	40m-shuttle runs
	4 x 6 x 5 s	2 x 12 x 30 s	4 x 6 x 5 s	2 x 12 x 30 s	4 x 6 x 5 s	2 x 12 x 30 s
**p.m.**	(r = 25 s; R = 5 min)	(r = 30 s; R = 3 min)	(r = 25 s; R = 5 min)	(r = 30 s; R = 3 min)	(r = 25 s; R = 5 min)	(r = 30 s; R = 3 min)
	all out	90% V_IFT_	all out	90% V_IFT_	all out	90% V_IFT_
	TL: 207 ± 64	TL: 270 ± 67	TL: 225 ± 67	TL: 257 ± 59	TL: 232 ± 68	TL: 290 ± 56

V_IFT_: final running speed obtained in the 30–15 Intermittent Fitness Test; r: passive recovery between intervals; R: passive recovery between series; TL: training load.

Example of training program: [40m-shuttle runs, 2 x 12 x 30 s, 90% V_IFT_, r = 30 s, R = 3 min] means that the subject had to run two series of 12 intervals at 90% V_IFT_ composed of 30 s passive recovery between intervals and 3 min passive recovery between series.

Example of training load calculation: [Session-RPE (9) x training duration (26 min)] = 234.

### Statistical analysis

All statistical analyses were performed by using SPSS (statistical software package version 18, SPSS Inc., Chicago, IL, USA) and Excel 2010 (Microsoft Corp., Redmond, WA, USA). Data are presented as mean ± SD and were tested for normal distribution using the Shapiro-Wilk-Test. Furthermore, 95% confidence interval (CI) is given. A two-factor (time, sex) repeated measure analysis of variance (ANOVA) was used to determine differences among markers of fatigue and recovery between testing days (pre, post_1_, and post_2_) as well as between male and female team sport athletes. Bonferroni post-hoc tests were used when the ANOVA main effect was significant. Those markers which were not normally distributed (CK, CRP) were tested using Friedman test. Wilcoxon tests were used when the Friedman test was significant. To allow a better interpretation of the results, the effect size Cohen`s *d* [27] (defined as [difference between the means]/SD) was calculated for all parameters between testing days. The thresholds for small, moderate, and large effects were 0.20, 0.50, and 0.80, respectively [27].

A 2 x 2 contingency table was used to evaluate the accuracy of the markers for the assessment of fatigue and recovery in comparison to the criterion measure (i.e., RSA). The table was composed of horizontal lines to indicate the presence or absence of fatigue (in accordance with changes in surrogate markers) and vertical lines to indicate the “true” condition of an athlete according to the criterion measure of fatigue. Diagnostic effectiveness (proportion of athletes correctly categorized by the surrogate marker), misclassification rate (proportion of athletes, who were incorrectly classified by the surrogate marker) and Youden`s index (ranges from 0 for a poor accuracy to 1.0 for an excellent accuracy of the surrogate marker) were calculated from the constructed table [28]. Finally, multiple regression analysis was used to assess relationships between changes in surrogate markers and criterion measure of fatigue and recovery. For all statistical analyses, level of significance was set at *p* < 0.05.

## Results

**Fig 2:**
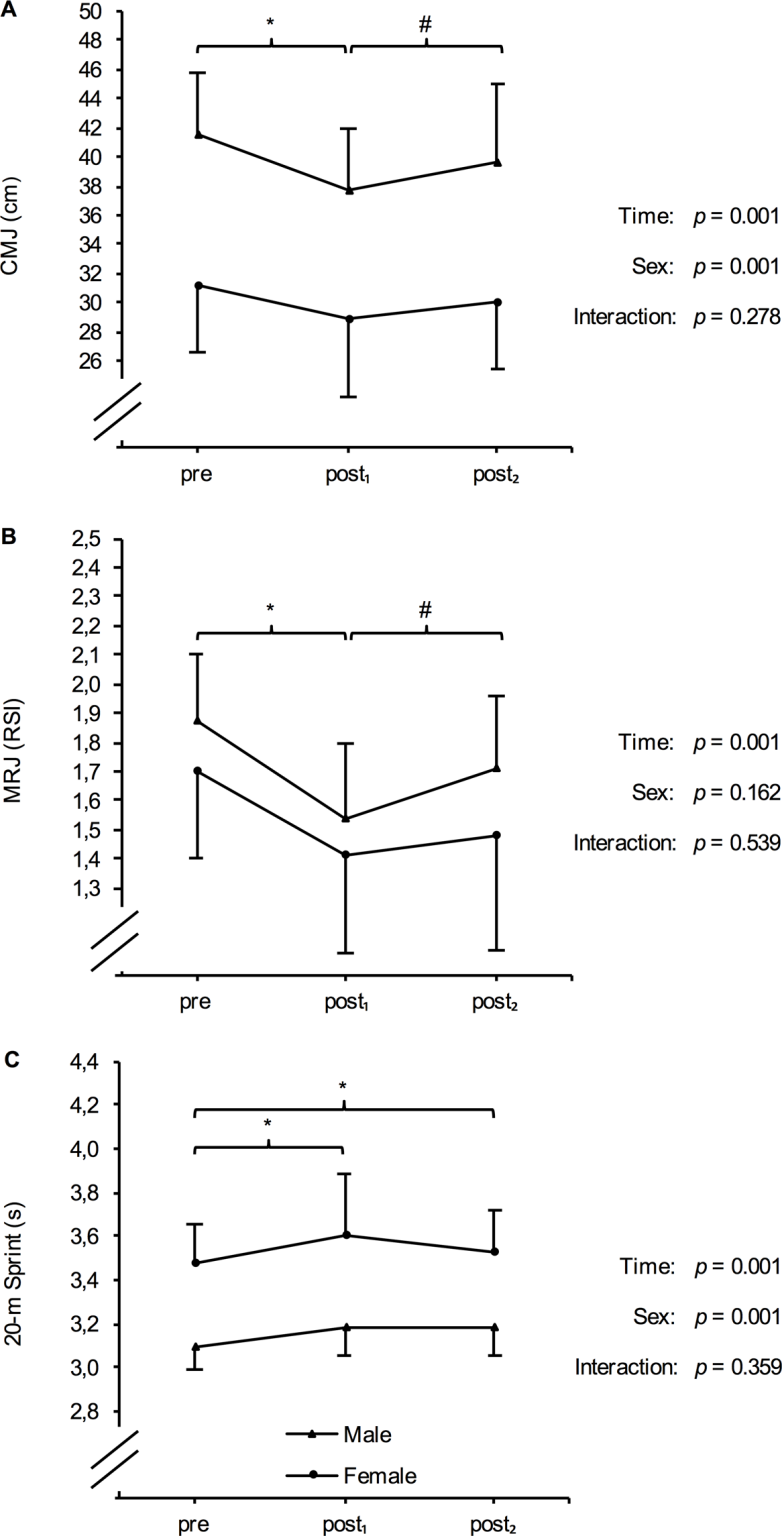
Mean (± SD) countermovement jump (CMJ) height (A), multiple rebound jumps (MRJ) performance (B) and 20-m sprint time (C) in males and females at baseline (pre), after a six-day high-intensity interval training program (post_1_), and following 72 h of recovery (post_2_). RSI = reactive strength index. *Significant difference compared to pre (*p* < 0.05). #Significant difference compared to post_1_ (*p* < 0.05).

**Fig 3:**
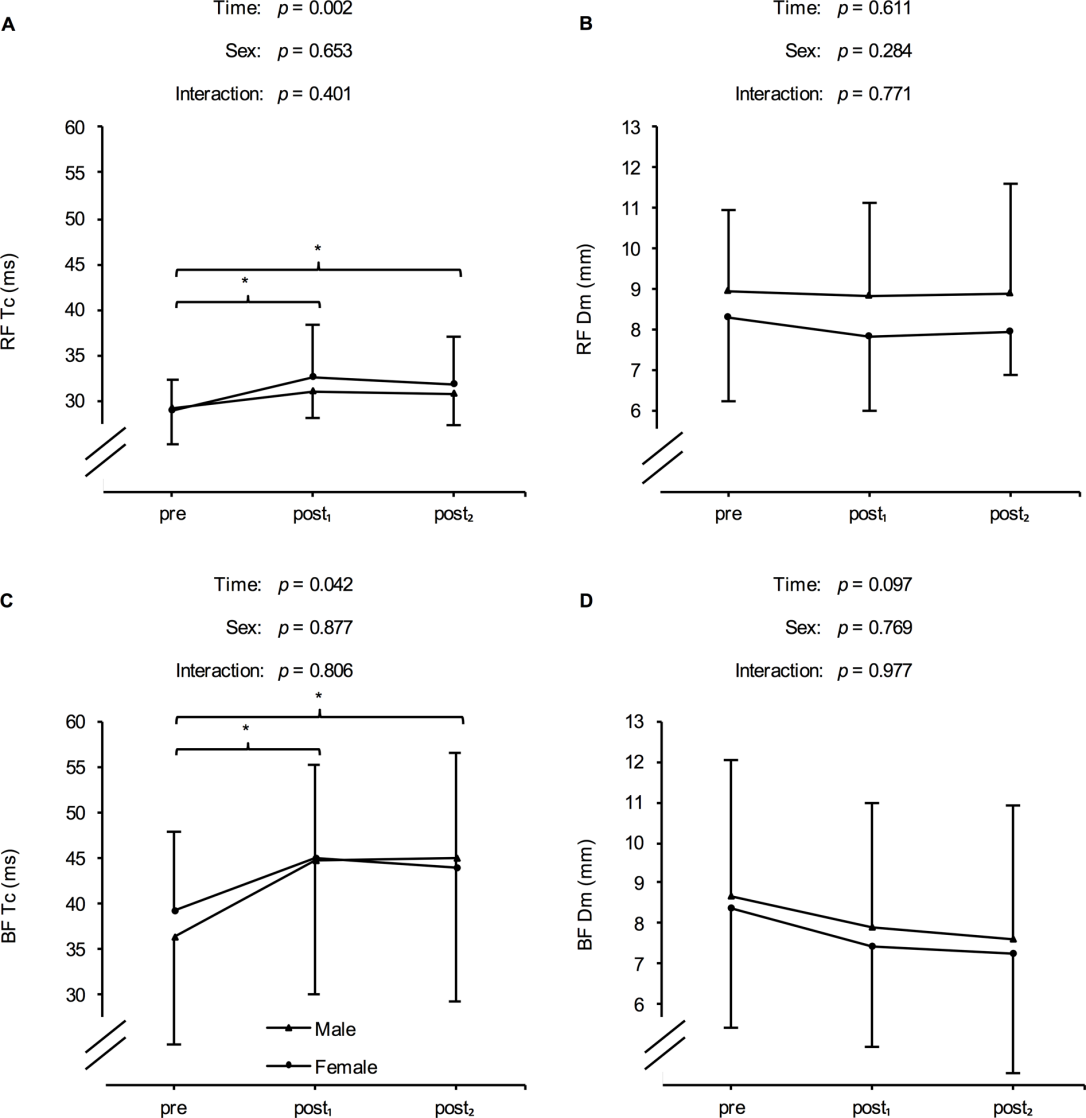
Mean (± SD) of the contraction time (Tc) and maximal radial muscle displacement (Dm) of the rectus femoris (RF) and biceps femoris (BF) in males and females at baseline (pre), after a six-day high-intensity interval training program (post_1_), and following 72 h of recovery (post_2_). *Significant difference compared to pre (*p* < 0.05). #Significant difference compared to post_1_ (*p* < 0.05).

**Fig 4:**
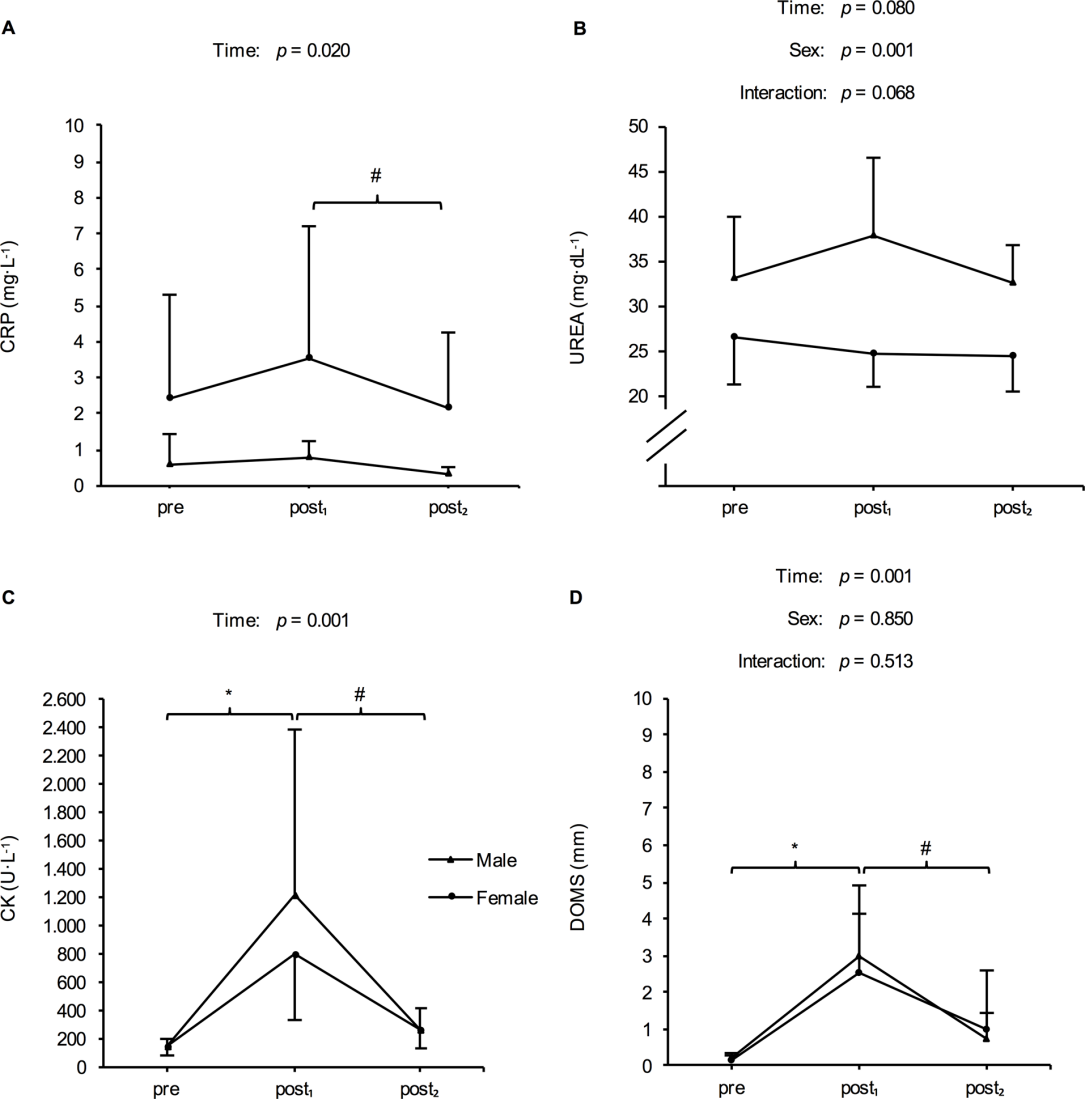
Mean (± SD) of the serum concentration of c-reactive protein (CRP), urea, and creatinkinase (CK) as well as of the rating of delayed onset muscle soreness (DOMS) in males and females at baseline (pre), after a six-day high-intensity interval training program (post_1_), and following 72 h of recovery (post_2_). *Significant difference compared to pre (*p* < 0.05). #Significant difference compared to post_1_ (*p* < 0.05).

No significant time x sex interaction (*p* = 0.566) but a significant main effect for time (*p* = 0.010) was found for RSA test performance. MV was significantly lower following the six-day training intervention (post_1_: 4.84 ± 0.56 m·s^−1^) than at baseline (pre: 5.02 ± 0.52 m·s^−1^) or after recovery (post_2_: 4.97 ± 0.56 m·s^−1^). The respective changes were -0.18 ± 0.13 m·s^−1^ (*p* = 0.001; effect size = -1.51) from pre to post_1_ and 0.12 ± 0.26 m·s^−1^ (*p* = 0.003; effect size = 0.53) from post_1_ to post_2_.Differentiated by sex, markers of fatigue and recovery are illustrated in Fig 2, Fig 3 and Fig 4. There were no significant time x sex interactions with respect to any of the determined markers. However, a significant main effect for time was found for CMJ, MRJ, and 20-m sprint performance, as well as for contraction time of the RF and BF, CK, CRP, and DOMS. For CMJ and MRJ performance, a significant decline and a return to baseline level after 72 h of recovery could be observed (Table 3). In addition, athletes demonstrated a significant increase in CK and DOMS following the training program and a significant decrease after the recovery period (Table 3). The HIIT-microcycle also induced a significant increase in 20-m sprint time and contraction time of the RF and BF at post_1_ compared to baseline values. However, these increases were not reversible between post_1_ and post_2_ (Table 3). Dm of the RF and BF, as well as CRP, and urea were not different at post_1_ and post_2_ compared to baseline values (Table 3).

**Table 3:** Markers of fatigue and recovery at baseline (pre), after a six-day high-intensity interval training program (post_1_), and following 72 h of recovery (post_2_) as well as percentage changes of performance and muscle contractile markers between testing days.

							Time	pre-post_1_		post_1_-post_2_		pre-post_2_	
	pre		post_1_		post_2_		*p*	%∆ ± CI	*d*	%∆ ± CI	*d*	%∆ ± CI	*d*
*Performance markers*													
CMJ (cm)	36.4±6.8	(33.4–39.4)	33.3±6.6*	(30.4–36.2)	34.8±6.9#	(31.8–37.9)	< 0.001	-8.4±2.9	-1.35	4.1±2.9	-0.68	-4.3±2.2	-0.79
MRJ (RSI)	1.79±0.28	(1.66–1.91)	1.48±0.30*	(1.34–1.61)	1.60±0.35#	(1.44–1.75)	< 0.001	-17.4±4.5	-1.60	6.5±4.5	-0.63	-10.9±5.3	-0.91
20-m Sprint (s)	3.28±0.24	(3.18–3.39)	3.40±0.30*	(3.26–3.53)	3.35±0.24*	(3.25–3.46)	< 0.001	3.4±1.8	-0.81	-1.2±1.6	-0.37	2.2±3.2	-0.65
*Muscle contractile markers*													
RF Tc (ms)	29.0±3.8	(27.3–30.7)	31.7±4.8*	(29.6–33.8)	31.2±4.6*	(29.2–33.2)	< 0.002	9.9±5.9	-0.72	-1.7±4.1	-0.17	8.2±6.0	-0.58
F Dm (mm)	8.6±2.1	(7.7–9.6)	8.3±2.2	(7.3–9.3)	8.4±2.1	(7.5–9.4)	< 0.611	-1.7±10.3	-0.17	2.1±6.2	-0.10	0.4±10.4	-0.11
BF Tc (ms)	37.7±10.7	(33.0–42.5)	44.9±12.9*	(39.2–50.6)	44.5±14.6*	(38.0–51.0)	< 0.042	28.7±24.9	-0.46	-8.1±21.5	-0.03	20.5±18.9	-0.59
BF Dm (mm)	8.5±3.3	(7.1–10.0)	7.7±2.9	(6.4–8.9)	7.4±3.2	(6.0–8.9)	< 0.097	-6.8±12.2	-0.39	-3.3±14.1	-0.10	-10.1±15.0	-0.46
*Biochemical markers*													
CK (U·L^−1^)	147±51	(125–170)	1010±887*	(617–1403)	269±134* #	(210–328)	< 0.001		-0.99		-0.95		-0.87
CRP (mg·L^−1^)	1.52±2.46	(0.33–2.70)	2.23±3.08	(0.74–3.71)	1.27±1.84#	(0.38–2.16)	< 0.020		-0.33		-0.66		-0.20
UREA (mg·dL^−1^)	29.6±7.2	(26.1–33.0)	30.9±9.4	(26.4–35.5)	28.2±5.9	(25.4–31.1)	< 0.080		-0.25		-0.46		-0.26
*Subjective markers*													
DOMS (mm)	0.2±0.1	(0.1–0.3)	2.7±1.8*	(2.0–3.5)	0.9±1.2#	(0.3–1.4)	< 0.001		-1.50		-1.44		-0.57

Parameters are shown as mean ± SD (95% confidence interval).

Cl: 95% confidence interval; *d*: Cohen`s d effect size; CMJ: countermovement jump; MRJ: multiple rebound jumps; RSI: reactive strength index; RF: rectus femoris; BF: biceps femoris; Tc: contraction time; Dm: muscle belly displacement; CK creatinkinase; CRP: C-reactive protein; DOMS: delayed onset muscle soreness.

*Significant difference compared to pre.

#Significant difference compared to post_1_.

Diagnostic effectiveness, misclassification rate and Youden`s index for surrogate markers of fatigue and recovery are shown in Table 4. None of the surrogate markers showed sufficient accuracy to discriminate athletes in a fatigued or recovered state in relation to RSA. Multiple regression analysis also revealed no significant correlations (p > 0.05) between changes in RSA and any of the surrogate markers.

**Table 4:** Accuracy of markers of fatigue and recovery in relation to the criterion measure.

	Diagnostic effectiveness (%)		Misclassification rate (%)		Youden`s Index	
	Δpre-post_1_	Δpost_1_-post_2_	Δpre-post_1_	Δpost_1_-post_2_	Δpre-post_1_	Δpost_1_-post_2_
*Performance markers*						
CMJ (cm)	63.6	60.0	36.4	40.0	0.01	0.20
MRJ (RSI)	68.2	60.0	31.8	40.0	0.08	0.20
20-m Sprint (s)	77.3	33.3	22.7	66.7	0.51	0.34
*Muscle contractile markers*						
RF Tc (ms)	68.2	40.0	31.8	60.0	0.38	0.21
RF Dm (mm)	50.0	66.7	50.0	33.3	0.04	0.30
BF Tc (ms)	54.5	40.0	45.5	60.0	0.03	0.23
BF Dm (mm)	50.0	60.0	50.0	40.0	0.11	0.20
*Biochemical markers*						
CK (U·L^−1^)	50.0	53.3	50.0	46.7	0.11	0.13
CRP (mg·L^−1^)	31.8	46.7	68.2	53.3	0.07	0.08
UREA (mg·dL^−1^)	30.0	46.7	70.0	53.3	0.10	0.00
*Subjective markers*						
DOMS (mm)	45.5	60.0	54.5	40.0	0.05	0.14

CMJ: countermovement jump; MRJ: multiple rebound jumps; RSI: reactive strength index; RF: rectus femoris; BF: biceps femoris; Tc: contraction time; Dm: muscle belly displacement; CK creatinkinase; CRP: C-reactive protein; DOMS: delayed onset muscle soreness.

## Discussion

The purpose of the present study was to investigate the accuracy of selected markers to reflect changes in fatigue and recovery in male and female team sport athletes during and after HIIT. The main finding of this study was that a six-day HIIT program induced significant changes in RSA, showing a temporary decline and a return to baseline level after 72 h of recovery. The decrease in RSA indicates that the training program induced a temporary state of fatigue. However, regular RSA testing for a routine assessment of fatigue and recovery may be unduly fatiguing and impractical for most athletes [29]. In this regard, the present study demonstrated that CMJ, MRJ, TMG Tc, CK and DOMS are potential markers of higher practicability and less demanding. This was evident in significant changes in these markers following the training period and after 72 h of recovery. However, due to an insufficient accuracy of these markers in differentiating between fatigued and recovered athletes, their responses to HIIT and their associations with fatigue and recovery appear to be highly individual. Since changes in markers of fatigue and recovery of males and females tended to be the same, these findings apply equally for both sexes.

Monitoring fatigue and recovery through measures of jump or sprint performance is recently utilized in the team sport environment due to its simplicity of administration, the minimal amount of additional fatigue induced, and its high reproducibility and validity [8, 9]. Therefore, we used the CMJ, the MRJ, and the 20-m linear sprint to monitor changes in the athlete`s neuromuscular function of the lower limbs during the six-day training intervention [22, 30]. In this study jump performance (i.e., jump height and jump efficiency) followed the changes in repeated sprint ability with a decrease in performance after the training period (CMJ: -8.4 ± 6.6%; MRJ: -17.4 ± 10.2%) and an increase of performance following the recovery period (CMJ: 4.9 ± 8.3%; MRJ: 8.5 ± 13.9%). Since the CV of the CMJ and MRJ performance was 3.7% and 4.0% respectively, the magnitude of changes can be considered to be of practical relevance. Linear sprint performance (CV = 1.8%) also showed a practically relevant decrease following the six-day training program of HIIT (3.4 ± 4.1%), but only tended to increase following the recovery period (-1.1 ± 3.4%).

Failure in the neuromuscular system responsible for altered performance can be explained by a combination of central and peripheral factors involving mechanisms from the central nervous system (e.g., impaired activation or reduced motivation) to exercise-related changes within the muscle fibers itself [9, 31, 32]. However, a decline in performance following exercise-induced fatigue has been demonstrated to be located peripherally (i.e., structural damage of muscle fibers, excitation-contraction coupling failure, redistribution of sarcomere length, impaired metabolism) rather than centrally [33, 34]. Since HIIT has the potential to induce muscle damage [33], it appears that the decreases in vertical jump height, jump efficiency (i.e., reactive strength index), and sprint performance may be related to repeated structural damage and inflammatory response of the muscle fibers caused by the HIIT program [29, 34]. It was shown that when muscle damage was induced through intense exercises, there were prolonged decreases in maximal force, ground reaction force, stretch-reflex sensitivity, muscle joint stiffness regulation and, thus, a reduction in jump and sprint performance [33]. Since the jump performance almost reached baseline levels and sprint performance showed a trend to increase following 72 h of recovery, these findings suggest that the CMJ, MRJ and 20-m sprint test may be potential tools to measure both fatigued and recovered neuromuscular function of team sport athletes following HIIT.

In addition to performance tests, measurements of selected blood markers under standardized conditions are proposed to monitor fatigued and recovered conditions [11]. In the practical team sport surrounding, routine blood parameters such as CK, CRP, and urea collected via capillary blood samples, are popular measures due to the simplicity of sample collection and analysis [7, 8, 12, 35]. In this study, CK reacted to the HIIT program, showing an average elevation of > 1000 U·L^−1^ after the training period and a decrease to almost baseline levels following the recovery period. However, no changes in CRP und urea could be observed between baseline, post_1_, and post_2_.

Serum CK activity mirrors the mechanical-muscular strain of the training since CK leak into the plasma from skeletal muscle fibers when they are damaged, including membrane damage and myofibrillar disruptions characterized by myofilament disorganization and loss of Z-disk integrity [9, 11]. Therefore, the elevated CK activity determined at post_1_ appears to support the explanation that damaged muscle fibers were partially responsible for the decline in performance. Similar to the present results, various studies with team sport athletes reported increased CK concentrations following intensified training or competition periods [12, 35–37]. The most likely explanation for the extremely high CK levels measured in this study was the characteristic of HIIT with its accelerations and decelerations as well as the changes of direction leading to high eccentric biomechanical strain on the working muscles, which in turn causes microinjuries of the musculoskeletal system and perceived muscle soreness [12, 33]. In this study, muscle soreness, which was measured subjectively by a VAS, followed the time course of CK activity (Table 3). DOMS increased following the training period and decreased after 72 h of recovery. Therefore, both the objective CK and subjective DOMS measures seemed to have the potential to identify HIIT-induced muscle damage associated with the fatigue and recovery observed in this study`s team sport athletes.

In this context, however, the high variability of measure of CK activity must also be taken into account [8]. Some athletes are non-responders due to a lower permeability of muscle cell membranes and only show small increases in CK activity [11]. Conversely, athletes with high percentages of fast twitch muscle fibers might tend to produce higher CK values [12]. Furthermore, sex could affect the magnitude of CK activity, which is due to a potentially higher CK content of men`s muscle than that of women`s muscle [9, 12, 38]. This assumption is supported by our data, since the mean CK concentration at post_1_ was 64.8% higher in the male compared to female participants (Fig 3). Therefore, athletes’ individual physical characteristics should be considered when using CK as an indicator of fatigue and recovery. One should also pay attention when solely using DOMS as a marker of fatigue and recovery. Since muscle function is impaired before soreness arises, and functional impairment may also persist when soreness has dissipated, this could lead to problems in an applied environment [33]. If solely the dissipation of muscle soreness is used as a signal to resume regular training, muscle function can be still in a weakened state and the risk of injury would be increased.

Since subsequent muscle damage is also linked to local inflammatory processes [9], the use of CRP may provide important additional information on the athlete’s status. However, despite an increase in CK activity in this study, no relevant changes in CRP could be determined following the HIIT-program (Table 3). In this context, Singh et al. [39] compared the effects of intermittent running, either with or without body ‘contact’, on muscle damage and inflammatory response. They demonstrated that both ‘contact’ and ‘non-contact’ training resulted in elevated serum CK, while CRP only increased following training with body ‘contact’. Since the addition of tackles to intermittent training further increased muscle damage following exercise, one can speculate that a certain degree of muscle damage requires ‘contact’ to significantly alter serum concentration of CRP. Based on the present results and due to the fact that potential interferences with inflammation are not directly related to muscle damage, it appears that CRP may not be a useful and specific enough marker for monitoring fatigue and recovery following HIIT.

This is also valid for urea, since serum concentrations were not altered at post_1_ and post_2_ compared to baseline values. Increased serum concentration of urea is a marker of enhanced protein catabolism and stimulated gluconeogenesis that results from high training volumes and increased energy consumption [12]. Since training volume during the HIIT-period was rather low (35 min per HIIT session; Table 2), no changes in urea and, thus, in the ‘anabolic-catabolic balance’ could be observed. This is in line with the findings by Coutts et al., [35] who reported unaltered urea serum concentrations following intensified training in rugby players.

Recent articles also recommend measures of muscle contractile properties as an effective method for detecting fatigue and recovery in athletes. In this context, TMG was introduced as an involuntary and non-invasive method to measure muscle contractile characteristics (i.e., Tc which is related to the speed of force generation, and Dm, which is representative of muscle tone and contractile force) [13]. Several studies have highlighted its usefulness for practitioners and researchers in detecting muscle damage and its recovery following various forms of exercises (i.e., eccentric exercise, endurance exercise, soccer) [13, 40–42]. For HIIT, the muscles affected most will be the extensor muscles of the knee joint (in the landing and take-off stages) and their antagonist muscles (traction in rear foot and leg recovery) [40]. Therefore, the muscle contractile characteristics of the RF and BF were measured through TMG in this study. Tc observed for both muscles significantly increased after the six-day training program and showed a trend for a decrease between post_1_ and post_2_. Dm was unaltered during all testing days.

Decreased Dm and increased Tc have been explained by a reduced efficiency of the excitation-contraction coupling, impairment in membrane conducting properties, and cellular structures destruction (i.e., peripheral fatigue) [42]. In this context, previous studies were able to demonstrate a decline in Dm and an increase in Tc when exercise-induced muscle damage (e.g., elevated CK activity and muscle soreness) was present [13, 42]. Since CK activity and DOMS were increased following the six-day HIIT-period, it can be concluded that Dm measured via TMG cannot be considered as a useful marker for monitoring fatigue and recovery following HIIT. On the other hand, due to an increase at post_1_ and a trend for a decrease at post_2_, Tc of the RF and BF may be a potential marker for monitoring fatigue and recovery.

As highlighted in the previous sections, measures of neuromuscular function, CK and DOMS are potentially useful markers for monitoring of team sport athletes during intensive training cycles. However, in relation to measures of sport-specific performance (i.e., RSA), which is demonstrably the most valid method for the assessment of fatigue and recovery, [8] none of the surrogate markers showed the ability to completely discriminate between fatigued and recovered athletes. Additionally, multiple regression analyses revealed that there were no relationships between changes in RSA and any of the surrogate markers. These findings indicate that responses of markers of fatigue and recovery to a given training stimulus are highly individual and variable, as already emphasized by Nèdèlec et al. [9] and Halson [8]. Additionally, Andersson et al. [37] showed, that the time course of the fatigue and recovery pattern differs significantly between various neuromuscular and biochemical markers. They demonstrated that CMJ performance, CK activity and muscle soreness were still changed 74 h following a football match, whereas sprint performance returned to baseline level already 5 h after the match. This could be a further explanation for the weak relationships between changes of surrogate markers and the criterion measure of fatigue. Consequently, accuracy of a single or combined use of CMJ, MRJ, 20-m sprint test, Tc, CK, and DOMS for the routine assessment of fatigue and recovery and their associations with sport-specific performance needs to be identified in practice for each athlete on an individual and longitudinal basis.

### Study limitations

First, although high V̇O_2max_ values were measured among the participants and most players were members of regional representative teams, the question remains whether the present results can be transferred to professional team sports at the international level. Effects might have been different with a group of high-level athletes. However, we have consciously refrained from recruiting elite players for this standardized research approach due to the reluctance of such populations to deviate from their normal training routine. Second, there was no control group to provide a baseline during the experimental period. In this regard, however, we have stated reliability data to indicate practically relevant changes in markers of fatigue and recovery. Third, the selection of markers that were evaluated in the present study might be considered a further limitation. There are especially some psychological markers (e.g., Recovery-Stress Questionnaire for Athletes [43]) that have been proposed in the literature as instruments to track the fatigue and recovery process and that have not been evaluated in the current investigation. However, the present study was not designed to analyze the highest possible number of markers of fatigue and recovery, but to evaluate a well-founded selection of practical tests that can be easily applied in team sports.

## Conclusions

The challenge for coaches and athletes is to determine the point at which intensive demands in training and competition lead to non-functional overreaching and may negatively affect the performance in upcoming competitions [8]. Therefore, routine assessment of fatigue and recovery is of importance to improve individual training prescription and to ensure competition readiness. To estimate changes in neuromuscular function following HIIT regardless of sex, this study was able to show that the power ability and reactive strength (i.e., CMJ, MRJ, 20-m sprint) in the lower body as well as Tc of the RF and BF are potentially useful markers.

However, in an applied environment, individual athletes respond differently to a given training stimulus, evidenced by the insufficient accuracy of the markers for monitoring fatigue and recovery in relation to the criterion measure. Therefore, surrogate markers should be assessed regularly in practice and with enough frequency to give the desired information to the athlete or coach. In this context, a possible recommendation for professional teams is to provide a fixed installation of a contact platform at the training ground to incorporate jump performance measurements as a daily routine. Also subjective assessment of DOMS using a visual analogue scale can be considered as a potential tool to identify team sport athletes who are susceptible to non-functional overload. In addition, CK as a routine blood marker may help to monitor the mechanical-muscular strain of HIIT. However, neither marker alone, nor specific group of markers significantly correlated with the criterion measure of fatigue. Therefore, a combination of the aforementioned markers should be used in practice in order to take into consideration all potential mechanisms that contribute to fatigue.

## Supporting Information

S1 Data Set (XLSX)
